# Prevalence of Radiological Chronic Pancreatitis and Exocrine Pancreatic Insufficiency in Patients with Decompensated Liver Disease: Is Fecal Elastase Useful in This Setting?

**DOI:** 10.3390/nu15020375

**Published:** 2023-01-11

**Authors:** Gemma Llibre-Nieto, Alba Lira, Mercedes Vergara, Meritxell Casas, Cristina Solé, José Ferrusquía-Acosta, Valentí Puig-Diví, Laia Grau-López, Josep Maria Barradas, Marta Solà, Mireia Miquel, Jordi Sánchez-Delgado

**Affiliations:** 1 Unitat Hepatologia, Servei Aparell Digestiu, Hospital Universitari Parc Taulí, Institut d’Investigació i Innovació Parc Taulí (I3PT), 08208 Sabadell, Spain; 2 Departament de Medicina, Universitat Autònoma de Barcelona, 08193 Bellaterra, Spain; 3 Hospital General de Granollers, 08402 Granollers, Spain; 4 Centro de Investigación Biomédica en Red de Enfermedades Hepáticas y Digestivas (CIBERehd), Instituto de Salud Carlos III, 28029 Madrid, Spain; 5 Unitat Gastroenterología, Servei Aparell Digestiu, Hospital Universitari Parc Taulí, Institut d’Investigacio i Innovació Parc Taulí (I3PT), 08208 Sabadell, Spain; 6 Estadística, Servei de Neurologia, Hospital Germans Trias i Pujol, 08916 Badalona, Spain; 7 Servei d’Infermeria, Unitat Hepatologia, Servei d’Aparell Digestiu, Hospital Universitari Parc Taulí, Institut d’Investigacio i Innovació Parc Taulí (I3PT), 08208 Sabadell, Spain; 8 Servei de Diagnòstic per la Imatge, Hospital Universitari Parc Taulí, Institut d’Investigacio i Innovació Parc Taulí (I3PT), 08208 Sabadell, Spain; 9 Departament de Medicina, Universitat de Vic–Universitat Central de Catalunya (UVic-UCC), 08500 Vic, Spain

**Keywords:** chronic pancreatitis, exocrine pancreatic insufficiency, decompensated cirrhosis, micronutrient deficiencies

## Abstract

Chronic alcohol consumption is a well-known etiological factor for both chronic pancreatitis (CP) and liver cirrhosis. However, there is discussion over how often these two entities are present together in the same patient. The main goal of our study is to establish the prevalence of CP and low fecal elastase (FE-1) in patients with decompensated liver disease (DLD). In addition, we aim to identify the demographic, epidemiological and clinical factors associated with EPI and CP in patients with decompensated liver cirrhosis. This was an observational single-center study including 119 consecutive patients hospitalized for acute decompensation of cirrhosis, mostly of alcoholic etiology. Patients underwent computed tomography (CT) or magnetic resonance imaging (MRI) to assess the radiological features of CP. We also performed two FE-1 tests and complete blood tests to assess the presence of exocrine pancreatic insufficiency (EPI) and nutritional status, including micronutrients. The results of our study show that 32 patients (26.9%) had low fecal elastase suggesting EPI and 11 (9.2%) had CP. Patients meeting radiological CP criteria had lower FE-1 than patients without CP. There were no statistically significant differences in micronutrient deficiencies according to the presence of CP or not. Likewise, we did not find any statistically significant differences in micronutrient deficiencies among patients with normal and low FE-1 indicative of EPI. FE-1 alone may not be suitable for assessing EPI in patients with acute DLD. Detecting co-existing pancreatic disease may be important in a subset of patients with DLD, when the FE-1 levels are significantly low, potentially suggestive of a pancreatic anomaly. Moreover, the clinical manifestations of EPI and CP are not useful in detecting CP in DLD patients. Likewise, CP cannot explain all causes of EPI in these patients.

## 1. Introduction

Chronic alcohol consumption is a well-known etiological factor for both alcoholic chronic pancreatitis (ACP) and alcoholic liver cirrhosis (ALC) and this risk directly correlates with the amount of alcohol consumed [1,2]. Only a minority of patients with chronic alcoholism will develop these diseases: about 3–5% will develop ACP, while only 2–3% will develop ALC [2,3]. In the case of ACP, the duration of alcohol consumption is shorter than in ALC; therefore, the diagnosis of ACP is usually made in younger patients than that of ALC [4].

The fact that ACP and ALC have a common etiological factor as important and recognized as chronic alcohol consumption suggests that the two conditions may coexist in some patients. However, there is discussion over how often these two entities are present together in patients with chronic alcoholism.

Existing studies present discordant results on this aspect; their different methodologies and evaluation of different parameters for each entity (clinical, functional, radiological or histopathological) make it difficult to compare them [4,5,6,7]. In addition, in clinical practice, the concurrence of ACP and ALC in the same patient is not very common, either because they are underdiagnosed or because they do not coexist. Finally, apart from chronic excessive alcohol consumption, the two entities do not share other risk factors [6].

Chronic pancreatitis (CP) is an inflammatory process of the pancreas that leads to the progressive destruction of the gland. The main symptom is abdominal pain, while steatorrhea and diabetes mellitus are clinical consequences that occur in the most advanced stages [8,9]. Specifically, exocrine pancreatic insufficiency (EPI) symptoms do not appear until approximately 90% of the exocrine function of the pancreas has been lost [10]. EPI manifestations include steatorrhea (often without diarrhea), dyspepsia, weight loss, vitamin deficiencies and altered bone metabolism [11]. Fat malabsorption leads to nutritional deficiencies, including those of fat-soluble vitamins (A, D, E, K) and possibly calcium, folic acid, magnesium, thiamine and zinc [12]. In addition, owing to vitamin D deficiency, patients with EPI are at a high risk of osteoporosis due to decreased bone mineral density [2,11]. The overall prevalence of bone disease (osteopenia and osteoporosis) in CP patients is greater than 60%, and one in four patients has established osteoporosis [13].

The diagnosis of EPI is challenging for clinicians since the symptoms and manifestations are not specific to this condition, but are also present in multiple gastrointestinal disorders, which can lead to late diagnosis or non-diagnosis. Key for diagnosis of EPI is a high clinical suspicion and identification of at-risk patients, such as those with CP [10,14]. It should also be noted that the probability of developing EPI secondary to CP also depends on the etiology, patients with ACP being at the greatest risk [15,16].

The diagnosis of EPI is based on the combination of symptoms, nutrient deficiencies and tests of pancreatic function. The most used and accessible test is FE-1, which has a specificity of 93% [17]. Given its high efficacy and non-invasiveness, this test could be considered the one of choice for screening patients with suspected EPI.

The commercial test used measures the concentration of fecal elastase in stool by enzyme-linked immunosorbent assay (ELISA) and determines the amount in feces of two isoforms of the human chymotrypsin-like elastase. Elastase is a very stable pancreatic enzyme in feces, easy to preserve, and correlates with the pancreatic secretion of various enzymes: elastase, lipase, amylase and trypsin. The only caution to be noted when collecting the sample is that it should be of solid consistency to avoid a false positive [18].

The main goal of our study is to establish the prevalence of CP and low FE-1 in patients with DLD, in a cohort of patients where the majority had ALC. In addition, we aim to identify the demographic, epidemiological and clinical factors associated with EPI—diagnosed on the basis of low FE-1—and CP in patients with decompensated liver cirrhosis.

## 2. Materials and Methods

### 2.1. Study Design

This was a prospective single-center cohort study conducted at Parc Taulí Hospital from October 2017 to December 2019.

### 2.2. Sampling and Inclusion Criteria

We included 119 consecutive patients with liver cirrhosis of any etiology admitted to our hospital due to decompensation (ascitic decompensation, infection, hepatic encephalopathy, alcoholic hepatitis, acute kidney injury or portal hypertensive bleeding). We excluded from the analysis all patients who had a previous diagnosis of pancreatic disease different from CP (cystic fibrosis, acute necrotizing pancreatitis and pancreatic tumors) and those who had undergone certain surgeries (pancreatic resection or gastrointestinal surgery) that could interfere with pancreatic function.

### 2.3. Sample Size Calculation

Few articles have evaluated the prevalence of ACP in patients with ALC, and we did not find any references on ACP prevalence in patients with DLD. The prevalence of ACP in patients with alcoholic liver disease was 16.2% in a recent meta-analysis [7]. The sample size of patients with decompensated liver cirrhosis was 106, which allowed us to estimate the prevalence of CP with a precision of ±7% and 95% confidence, assuming a prevalence of 16% of the variable of interest.

### 2.4. Data Collection and Measurements

Patients were identified with a numerical code to ensure the confidentiality of their personal data after they had been properly informed and given signed consent. The following variables were obtained at the time of inclusion: demographic data (age and sex); smoking and alcohol intake; presence of diabetes; anthropometric parameters (weight and height for body mass index calculation); reason for admission; blood test results: hemogram, bilirubin, international normalized ratio, renal function, albumin, prealbumin, total protein, cholesterol (high-density lipoprotein (HDL) and low-density lipoprotein (LDL)), triglycerides, fat-soluble vitamins (A, D, E, K), water-soluble vitamins (B1, B6, B9 or folic acid, B12 and C) and trace elements (calcium, phosphorus, zinc, magnesium, copper, iron and ferritin); and cirrhosis etiology: alcoholic, hepatitis C virus (HCV), hepatitis B virus (HBV) or metabolic-associated fatty liver disease (MAFLD). The vitamin forms measured were the following: retinol (vitamin A), thiamine diphosphate (Vitamin B1), pyridoxal phosphate (vitamin B6), cyanocobalamin (vitamin B12), folic acid (vitamin B9), ascorbic acid (vitamin C), 25-hydroxicholecalciferol (vitamin D), alpha-tocopherol (vitamin E) and phylloquinone (vitamin K).

We also recorded the presence of hepatic complications (ascitic decompensation, infection, hepatic encephalopathy, alcoholic hepatitis, acute kidney injury and portal hypertensive bleeding).

We included only patients with alcoholic hepatitis who required admission because they met at least one of the following hospital admission criteria: hepatic encephalopathy, bilirubin levels >2 mg/dL, prothrombin time >1.36, acute kidney injury, ascites or significant social problems. We defined acute kidney injury (AKI) according to the position paper of the International Club of Ascites: an increase in serum creatinine ≥0.3 mg/dL within 48 h or a ≥50% increase in serum creatinine from baseline, occurring in the previous seven days [19].

The severity of the liver dysfunction was assessed using the Child–Pugh classification system and the MELD score (Model for End-stage Liver Disease), in which a higher score indicates more severe liver dysfunction. During hospital stay, we took two stool samples from each patient to measure FE-1 (ScheBo BiotechAG Germany Stool Test). The nurses who collected the samples and the laboratory staff who analyzed them were aware that liquid samples should be rejected to avoid false positives. At the time of admission, patients underwent a clinical questionnaire regarding the symptoms they experienced at home that could be suggestive of CP with EPI: diarrhea (defined as ≥3 stools/day), steatorrhea and recurrent epigastric pain.

To assess CP features, patients underwent a CT scan or an MRI according to clinical indication and scanner availability.

### 2.5. Definition of Variables

We defined chronic pancreatitis as the presence of at least one of the following radiological criteria [20]:

– Glandular atrophy not explained by age.
– Irregularities or ectasia of the duct of Wirsung.
– Presence of calcifications.

We considered patients to have EPI if they had two stool samples with fecal elastase <200 μg/g. If one or both of the values were >200 μg/g, we considered this to mean the absence of EPI [21].

### 2.6. Statistical Methods

Descriptive analysis was performed. Continuous variables were described using mean and standard deviation (SD) or median and interquartile range, as appropriate. For categorical variables, absolute numbers and percentages were calculated with their 95% confidence intervals. We used the *t*-test for continuous variables. For categorical variables, Fisher’s test was used when there were fewer than five categories for the variable in question, while we used the chi-squared test if there were five or more categories. We collected and analyzed data using SPSS 23.0. In all cases, statistical significance was reached when the *p*-value was less than 0.05.

### 2.7. Ethics

Patients were recruited consecutively as they were admitted to our hospital due to cirrhosis decompensation. Once the inclusion criteria were met, patients were identified with a numerical code to ensure the confidentiality of their personal data after they had been properly informed and given signed consent.

The design, procedures and goal of the study were approved by the corresponding ethics review board (Clinical Research Ethical Committee of Parc Taulí Hospital, Ref.: 2017536), ClinicalTrial gov identifier: NCT03236038. As previously stated, the participants were given information about the content of the study and signed a consent form before their inclusion. The study complied with the ethics set out in the Declaration of Helsinki. Confidentiality was preserved in agreement with the existing Spanish Data Protection Law (15/1999).

## 3. Results

### 3.1. Patient Characteristics

A total of 119 patients with DLD were included. Baseline patient characteristics are shown in Table 1. The mean age was 62.4 ± 10.2 years and 78.2% were men.

A total of 110 patients underwent a radiological investigation: 104 had a CT scan and 6 an MRI.

The etiology of liver cirrhosis was alcoholic in 92% of the patients (84.9% alcoholic only vs. 6.7% alcoholic and HCV). Of these, 57.8% still consumed alcohol. A total of 43.6% of the patients were active smokers and 35.3% had diabetes mellitus. Eleven patients were in Child–Pugh class A (9.2%), 68 were in Child–Pugh class B (57.1%) and 40 were in Child–Pugh C (33.6%). The mean MELD score was 15.9 ± 6.

### 3.2. Chronic Pancreatitis and Decompensated Liver Disease

The vast majority of our patients had alcoholic cirrhosis (92%): 84.9% were of alcoholic etiology only and 6.7% were caused by alcohol and HCV. We found that 9.2% (11 of the 110 patients with a radiological investigation) met the established radiological criteria for CP.

There were no significant differences in the prevalence of CP in DLD according to age, sex, type of cirrhosis decompensation, etiology of cirrhosis, severity of liver disease, current alcohol consumption, active smoking or presence of diabetes mellitus. These results are summarized in Table 2.

### 3.3. Chronic Pancreatitis and EPI

The results of our study show that 32 patients (26.9%) had two low FE-1 results (<200 µg/g) indicative of EPI. Of these patients, 81.2% had an alcoholic etiology of liver disease and 18.8% had a non-alcoholic etiology.

Patients meeting the radiological CP criteria had a lower FE-1 than patients without CP: seven (21.9%) vs. four (5.1%), *p* = 0.013. There were no significant differences in relation to the presence of recurrent epigastric pain, diarrhea, steatorrhea or micronutrient deficiencies. There were also no significant differences in the presence of low fecal elastase indicative of EPI according to age, sex, type of cirrhosis decompensation, etiology of cirrhosis, severity of liver disease, current alcohol consumption, active smoking or presence of diabetes mellitus. These results are summarized in Table 3.

As for the clinical manifestations of EPI at home according to the clinical questionnaire, for the entire cohort (119 patients), 32.8% reported having diarrhea, 26.9% reported steatorrhea and 12.6% reported having recurrent epigastric pain. There were no differences in the clinical EPI data among those with normal and low FE-1.

### 3.4. Micronutrient Deficiencies and Low Fecal Elastase

In the entire cohort of patients with decompensated cirrhosis, the most frequently found deficiencies were for fat-soluble vitamins: 91.6% of patients had a vitamin A deficiency and 94.4% had a vitamin D deficiency. With regard to water-soluble vitamins, 64.9% and 33.8% of patients had vitamin B6 and vitamin C deficiency, respectively. For trace elements, 84.9% had zinc deficiency, 38.7% had iron deficiency and 34.5% had phosphorus deficiency (Table 1).

There were no statistically significant differences in micronutrient deficiencies according to the presence of CP. We also did not find any statistically significant differences in micronutrient deficiencies among patients with normal or low fecal elastase indicative of EPI. We used a *t*-test for continuous variables. For categorical variables, Fisher’s test was used when there were fewer than five categories for the variable in question, while we used the chi-squared test if there were five or more categories.

## 4. Discussion

This study is the first to be conducted in patients with an acute decompensation of liver disease and admitted to hospital during the assay, which makes the very-well-controlled collection of samples possible.

In our study, we found a prevalence of radiological CP of 9.2%, which is not insignificant but lower than expected. The prevalence of CP in patients with alcoholic liver disease has been described in a recent metanalysis: 16.2% (95% CI 10.4–24.5) overall and 15.5% (95% CI 8.0–27.7) when data were limited to clinical studies [7].

The diagnosis of CP is usually made on the basis of clinical and radiological features. For the radiological diagnosis, CT, MRI or endoscopic ultrasound are used. CT and MRI have similar sensitivity (75% vs. 78%) and specificity (91% vs. 96%), whereas endoscopic ultrasound has a sensitivity of 81% and a specificity of 90% [20,22]. However, endoscopic ultrasound has the disadvantage of being an invasive test.

It should be taken into account that, of this 9.2% of patients with CP, not all of them present altered classical diagnostic tests. Specifically, 63.6% have a low FE-1 compared to 25.2% in patients with a normal pancreas (*p* = 0.02).

In our study, none of the 11 patients with CP had been previously diagnosed with episodes of acute pancreatitis (neither acute pancreatitis alone nor recurrent acute pancreatitis). Additionally, only 2 of the 11 patients reported recurrent epigastric pain. Of the nine remaining patients, six presented recurrent abdominal discomfort that was not suggestive of pancreatic pathology, but rather was attributable to other etiologies (ascites, dyspepsia, irritable bowel syndrome, among others). The other three did not present any abdominal discomfort.

As for steatorrhea, it was only described in 4 of the 11 patients with CP and it was also described in 27% of patients with a normal pancreas. We believe that this can be justified since DLD patients usually present very liquid stools due to the use of laxatives and the active consumption of alcohol (present in more than 50%) that accelerates peristalsis. This is the reason why we think that steatorrhea would not be a useful sign to assess the presence or absence of EPI in DLD patients.

Regarding the patients with CP and DM, it should be remarked that 9 of the 11 patients had been diagnosed with DM prior to admission. Only the remaining two were diagnosed during hospital admission. In any case, it should be noted that, during admission, the vast majority presented a poor glycemic control with a markedly elevated insulin requirement. However, we cannot determine whether DM in these patients could be a complication of cirrhosis, secondary to pancreatic pathology, or due to other causes.

Additionally, several studies agree that male sex is an important risk factor for developing CP, as men with acute pancreatitis have been shown to have a higher risk of progression to CP than women [16,23]. In our study, of the patients with CP, 80% were men. In addition, smoking accelerates the progression to CP and more advanced stages [24]. In our study, of the patients with CP, 60% were current smokers.

On the other hand, the prevalence of EPI in the general population is unknown. EPI is mostly associated with advanced stages of CP. Among patients with CP, 60–90% will develop EPI within 10–12 years from diagnosis [25]. Normally, patients with advanced CP are managed in tertiary centers, while patients with early CP are managed in primary care. It has been reported that the prevalence of EPI in these patients with early stage disease managed in primary care is around 18.7% [26].

In our study, a high percentage of DLD patients (26.9%) presented low FE-1—which is suggestive of EPI– for reasons other than CP. These findings suggest that FE-1 may not be an appropriate method to study EPI in patients with acute decompensation of liver disease and that CP may not explain all causes of EPI in these patients. It would therefore be advisable to search for other, less common causes of EPI in addition to CP, such as celiac disease, inflammatory bowel disease or cystic fibrosis, or find another diagnostic test with a lower false positive rate. The presence of EPI not attributable to CP could not be explained by diabetes mellitus, since there was a similar distribution of DM among those with low and normal fecal elastase values (31.3% vs. 36.8%, *p* = 0.66).

Regarding the interpretation of results, concentrations of <200 μg/g are considered pathological and <100 μg/g are considered severe EPI. According to these definitions, we found that 34.5% of the patients with EPI fell into the severe category.

Moreover, we found a higher prevalence of low FE-1 in patients with CP than in patients without CP (21.9% vs. 5.1%, *p* = 0.013). We did not find a significant difference in the prevalence of low FE-1 between the cohorts with alcoholic and non-alcoholic cirrhosis.

Taking these results into account, we could say that measuring FE-1 levels may help to identify a subset of DLD patients with a pancreatic pathology. However, it should be noted that low FE-1 values by themselves do not imply that DLD patients necessarily suffer from a pancreatic pathology.

In addition, in clinical practice, the co-occurrence of DLD and CP in the same patient is not very common. Actually, it seems that these are two entities with different etiopathogenesis and prognosis that share alcohol as a risk factor [6]. For this reason, some authors tried to identify other clinical factors responsible for the co-occurrence of DLD and CP.

Thus, Tan et al. conducted a cohort study and concluded that significant risk factors for CP in alcoholic liver disease patients included smoking and multiple bouts of acute pancreatitis [27].

Similarly, Chand et al. found that the number of pancreatitis recurrences was a significant factor associated with liver disease. Additionally, they showed that, for the same number of pancreatitis recurrences, the frequency of liver disease was 1.7 times significantly higher in patients with CP than in patients with recurrent pancreatitis who did not have CP (*p* < 0.0001). Moreover, they found that 15% of patients with cirrhosis had common bile duct pathologies or obstruction of the duodenum. Then, they suggested to discuss the possibility of the involvement of the common bile duct as a mechanistic cause leading to liver disease. In the same way, they found that there were patients with non-alcoholic pancreatitis who developed cirrhosis and, therefore, may still need to be screened for liver disease [28].

Additionally, Lankisch et al. estimated that <5% of those with high alcohol intake develop CP, which suggests that there are other predisposing factors in CP in addition to alcohol [3]. This correlates with our findings, since we found a prevalence of CP of 9.2% in a cohort of patients with mostly alcoholic liver disease.

These findings suggest that there is a low concurrence of CP and liver cirrhosis in the same patient and that these diseases evolve in different ways. These findings also correlate with the results found by Nakamura et al. [4]. They stated that CP and LC are associated with different lifestyle factors and that there is not an association between the severity of these two entities. Aparisi et al. [6] found an inverse correlation between indexes of pancreatic and liver function in the same patient, stating that the etiopathogenesis is different, although chronic alcoholism is a necessary factor in both.

In 2014, Rabih et al., using the ^13^C-mixed triglyceride breath test, found that a higher proportion of patients with chronic alcoholic liver disease had EPI compared with patients with chronic liver disease of any other etiology (55.2% vs. 16.7%, *p* < 0.001) [29]. They also found that EPI was more common in patients without cirrhosis (70%) than in patients with cirrhosis (46.2%, *p* = 0.017).

Aparisi et al. found a lower prevalence of EPI in patients with cirrhosis (7%) compared with asymptomatic alcoholic patients (14.8%) [6]. They assessed fecal elastase to rule out EPI. Hayakawa et al. reported a negative correlation between liver and pancreatic function in patients with ALC [30], and observed increased pancreatic secretion in patients with alcoholic liver disease.

It is worth noting, then, that although there is a variety of studies that try to shed light on the relationship and co-occurrence between CP and DLD, the evidence remains poor and some results are contradictory.

Another topic that should be discussed is that the presence of EPI did not correlate with micronutrient deficiency or severity of liver insufficiency. Joker-Jensen et al. analyzed the prevalence of micronutrient deficiencies in patients with CP [31]. They found that 22% of patients had vitamin D deficiency, 20% had zinc deficiency, 17% had magnesium deficiency, 10% had vitamin A deficiency and 7% had vitamin E deficiency. This is important because the prevalence of micronutrient deficiencies in our sample were much higher (94.4%, 84.9%, 12.6%, 91.6% and 15%, respectively) and is attributable to the decompensated liver disease, meaning that we could not assess the micronutrient deficiencies derived from EPI.

It is well-known that DLD patients present sarcopenia in relation to an imbalance between protein synthesis and breakdown, due to an increased catabolism and energy expenditure. Additionally, they have diminished vitamin reserves with respect to the general population, usually due to hepatic dysfunction, low dietary intake, low absorption and increased catabolism. In addition, malabsorption and maldigestion and the use of diuretics contribute to micronutrient deficiencies [32,33]. This is the reason why we cannot know if there is a percentage of nutrient deficit that could be attributed to a chronic pancreatic pathology in some DLD patients.

Regarding the limitations of our study, one aspect that should be taken into account is that the radiological criteria for CP are based on cohorts of patients with advanced pancreatic disease, so the existence of incipient disease in our patients cannot be completely excluded. Another issue worthy of comment is that we used CT and MRI to assess the radiological signs of CP. Had we used an invasive technique such as endoscopic ultrasound, we may have diagnosed more patients as it has a greater sensitivity, especially in earlier stages.

Another potential limitation of this study is the reliability of fecal elastase as a diagnostic test for EPI. EPI could be diagnosed through invasive methods, such as the secretin stimulation test, which has a higher sensitivity and specificity but requires the insertion of a tube into the small intestine to collect pancreatic secretions. In contrast, the fecal fat test is non-invasive but is very difficult to perform during hospital admission as it requires a specific diet and stool collection over a period of days. The major drawbacks of fecal elastase are its low sensitivity in the early stages of the disease and some possible false positives if the samples are of liquid consistency. As already described in the methods, we made sure loose or watery samples were discarded.

Finally, it should be borne in mind that the vast majority of our patients (92%) had alcoholic cirrhosis. Therefore, the findings may not be representative of other etiologies of liver disease.

In summary, there is little evidence of an association between pancreatic and hepatic function in patients with chronic alcohol consumption, especially those with cirrhosis. The latest evidence indicates that CP and LC are two distinct entities in terms of etiopathogenesis and outcomes, and that the only shared risk factor is alcohol.

More studies should be conducted to clarify this issue, especially with regard to the presence of incipient pancreatic disease in patients with liver cirrhosis.

## 5. Conclusions

In patients with DLD and admitted to hospital, we found a prevalence of CP of 9.2% and a prevalence of low fecal elastase suggesting EPI of 26.9%. Our findings reveal that FE-1 alone may not be a suitable method to assess EPI in patients with an acute decompensation of liver disease, and that CP may not explain all causes of EPI in these patients. Detecting co-existing pancreatic diseases may be important in a subset of patients with DLD, when FE-1 levels are significantly low, potentially suggestive of a pancreatic anomaly. The typical clinical manifestations of CP and EPI (recurrent epigastric pain, diabetes mellitus and steatorrhea) are not useful to discriminate which patients have CP in decompensated cirrhosis. Micronutrient deficiencies are very frequent in patients with decompensated liver cirrhosis and their presence does not indicate CP or IPE.

## Figures and Tables

**Table 1:** Summary of the baseline characteristics for all included patients (*n* = 119).

Variable	Value
Age (years)	62.4 ± 10.3
Sex	
Male	93 (78.2%)
Female	26 (21.8%)
Current smoker	51 (43.6%)
*Etiology of cirrhosis*	
Alcohol	101 (84.9%)
Alcohol and HCV	8 (6.7%)
Others (HCV, HBV, MAFLD, cryptogenic)	10 (11.9%)
Current alcohol consumption	67 (57.8%)
Diabetes mellitus	42 (35.3%)
*BMI categories*	
Underweight (<18.5)	2 (1.7%)
Normal weight (18.5–24.9)	31 (26%)
Overweight (25–29.9)	45 (37.8%)
Obese (30–39.9)	27 (22.6%)
Extremely obese (>40)	8 (5.8%)
*Child–Pugh class*	
A	11 (9.2%)
B	68 (57.1%)
C	40 (33.6%)
MELD score	15.9 ± 6
*Decompensation of cirrhosis*	
Ascites	97 (81.5%)
Encephalopathy	28 (23.5%)
Portal hypertensive bleeding	17 (14.3%)
Acute kidney injury	19 (16%)
Alcoholic hepatitis	17 (14.3%)
Non-SBP infection	40 (33.6%)
SBP	11 (9.2%)
Low fecal elastase (<200 μg/g)	32 (26.9%)
Chronic pancreatitis (*n* = 110)	11 (9.2%)
Diarrhea	39 (32.8%)
Steatorrhea	32 (26.9%)
Recurrent epigastric pain	15 (12.6%)
*Vitamin deficiencies*	
Vitamin A (<0.3 mg/L)	109 (91.6%)
Vitamin D (<30 ng/mL)	101 (94.4%)
Vitamin E (<5 µg/mL)	16 (15%)
Vitamin K (<0.13 µL/L)	3 (3.4%)
Vitamin B1 (<2 µg/dL)	3 (3.4%)
Vitamin B6 (<23 nmol/L)	50 (64.9%)
Vitamin B9 (<2 ng/mL)	5 (4.2%)
Vitamin B12 (<150 pg/mL)	0
Vitamin C (<0.4 mg/dL)	26 (33.8%)
*Trace element deficiencies*	
Calcium (<8.8 mg/dL)	6 (5%)
Phosphorous (<2.7 mg/dL)	41 (34.5%)
Magnesium (<1.6 mg/dL)	15 (12.6%)
Copper (<70 µg/dL)	18 (15.1%)
Iron (<60 µg/dL)	46 (38.7%)
Ferritin (<30 ng/mL)	5 (4.2%)
Zinc (<68 µg/dL)	101 (84.9%)

Data are mean (SD) for quantitative variables and n (%) for qualitative variables. MELD: Model for end-stage liver disease; SBP: Spontaneous bacterial peritonitis.

**Table 2:** Chronic radiological pancreatitis vs. normal pancreas (*n* = 110).

Variable	Chronic Pancreatitis 11 (9.2%)	Normal Pancreas 99 (90.8%)	*p*-Value
Age (years)	65 ± 13.6	62.4 ± 10	0.71
Female	2 (20%)	24 (24%)	0.77
Low fecal elastase (<200 µg/g)	7 (63.6%)	25 (25.2%)	0.02
Diarrhea	2 (20%)	35 (35%)	0.4
Steatorrhea	4 (40%)	27 (27%)	0.38
Recurrent epigastric pain	2 (20%)	12 (12%)	0.47
Current smoker	6 (60%)	41 (41.8%)	0.39
Current alcohol consumption	4 (40%)	54 (55.7%)	0.5
Alcoholic cirrhosis	8 (80%)	84 (84%)	0.66
Child–Pugh Class			
A	2 (19%)	9 (9.1%)	0.37
B	4 (36%)	60 (60.6%)	0.37
C	5 (45%)	30 (30.3%)	0.37
MELD score	15.4 ± 6.8	15.2 ± 5.5	0.3
Diabetes mellitus	4 (40%)	38 (38.3%)	0.8
BMI	25.8 ± 4.03	27.6 ± 6.5	0.44
Prealbumin (20–40 mg/dL)	8.12 ± 3.4	9.03 ± 4.16	0.7
Albumin (34–48 g/L)	31.7 ± 5.1	30 ± 5.9	0.42
Cholesterol (125–200 mg/dL)	103.28 ± 28.4	124 ± 53.4	0.38
Triglycerides (50–200 mg/dL)	78 ± 20.5	95.4 ± 55.4	0.83
Decompensation of cirrhosis			
Ascites	8 (80%)	89 (89.8%)	0.87
SBP	0	11 (11%)	0.26
Encephalopathy	2 (20%)	26 (26.2%)	0.72
Portal hypertensive bleeding	1 (10%)	16 (16.1%)	0.67
Non-SBP infection	5 (50%)	33 (33%)	0.31
Alcoholic hepatitis	1 (10%)	15 (15%)	0.67
Hepatorenal syndrome	0	13 (13%)	0.6
Micronutrient levels and deficiencies			
Vitamin A levels (0.3–1 mg/L)	0.11 ± 0.1	0.13 ± 0.12	0.7
Vitamin A deficiency (<0.3 mg/L)	10 (100%)	90 (91.8%)	0.44
Vitamin D levels (>30 ng/dL)	6.6 ± 4.06	9.4 ± 6.8	0.17
Vitamin D deficiency (<30 ng/dL)	8 (80%)	86 (96%)	0.66
Vitamin E levels (5–20 µg/dL)	6.3 ± 3.2	9.3 ± 4.1	0.012
Vitamin E deficiency (<5 µg/dL)	3 (30%)	12 (12.2%)	0.14
Vitamin K levels (0.13–1.50 µg/L)	0.38 ± 0.19	0.85 ± 0.82	0.11
Vitamin K deficiency (<0.13 µg/L)	0	2 (2.7%)	0.68
Vitamin B1 levels (2–7.2 µg/dL)	5.2 ± 4.3	3.9 ± 1.39	0.85
Vitamin B1 deficiency (<2 µg/dL)	1 (16.7%)	1 (1.6%)	0.17
Vitamin B6 levels (23–173 nmol/L)	20.54 ± 19.9	24 ± 18.67	0.59
Vitamin B6 deficiency (<23 nmol/L)	6 (60%)	57 (60%)	0.62
Vitamin B9 levels (2–14.54 ng/mL)	7.4 ± 6.2	6.8 ± 3.94	0.93
Vitamin B9 deficiency (<2 ng/mL)	1 (11.1%)	4 (5.2%)	0.47
Vitamin B12 levels (150–695 pg/mL)	1129.2 ± 837.6	1062.8 ± 603.74	0.75
Vitamin B12 deficiency (<150 pg/mL)	0	0	1
Calcium (8.8–10.2 mg/dL)	9.2 ± 0.4	9.27 ± 0.54	0.24
Calcium deficiency (<8.8 mg/dL)	0	6 (6.6%)	0.52
Phosphorous (2.7–4.5 mg/dL)	3.1 ± 0.76	2.7 ± 0.8	0.55
Phosphorous deficiency (<2.7 mg/dL)	2 (22.2%)	36 (40%)	0.47
Magnesium (1.6–2.6 mg/dL)	1.1 ± 0.26	1.8 ± 0.4	0.47
Magnesium deficiency (<1.6 mg/dL)	0	13 (17.1%)	0.34
Copper (70–140 µg/dL)	114.2 ± 26.4	91.5 ± 35.9	0.28
Copper deficiency (<70 µg/dL)	1 (14.3%)	16 (20%)	0.71
Iron (60–158 µg/dL)	105.3 ± 95	74.6 ± 55.2	0.36
Iron deficiency (<60 µg/dL)	3 (30%)	38 (40.4%)	0.74
Ferritin (30–400 ng/mL)	164.9 ± 121.4	303.8 ± 321.1	1
Ferritin deficiency (<30 ng/mL)	1 (10%)	4 (4.3%)	0.41
Zinc (68–120 µg/dL)	53.3 ± 2.3	43.8 ± 17.2	0.04
Zinc deficiency (<68 µg/dL)	8 (88.9%)	85 (90.4%)	0.88

Data are mean ± SD for quantitative variables and *n* (%) for qualitative variables. MELD: Model for end-stage liver disease; SBP: Spontaneous bacterial peritonitis; BMI: Body mass index.

**Table 3:** Exocrine pancreatic insufficiency vs. normal fecal elastase (*n* = 119).

Variable	Low Fecal Elastase 32 (26.9%)	Normal Fecal Elastase 87 (73.1%)	*p*-Value
Age (years)	62.6 ± 10.5	62.3 ± 10.2	0.85
Female	10 (31.3%)	16 (18.4%)	0.14
Chronic pancreatitis	7 (21.9%)	4 (5.1%)	0.013
Diarrhea	5 (15.6%)	34 (39.1%)	0.016
Steatorrhea	9 (28.1%)	23 (26.4%)	0.85
Recurrent epigastric pain	4 (12.5%)	11 (12.6%)	0.98
Current smoker	12 (37.5%)	39 (45.9%)	0.71
Current alcohol consumption	15 (46.9%)	52 (61.9%)	0.2
Alcoholic cirrhosis	26 (81.3%)	75 (86.2%)	0.56
Child–Pugh Class			
A	4 (12.5%)	7 (8%)	0.75
B	18 (56.3%)	50 (57.5%)	0.75
C	10 (31.3%)	30 (34.5%)	0.75
MELD score	15.9 ± 5.1	16 ± 6.3	0.82
Diabetes mellitus	10 (31.3%)	32 (36.8%)	0.66
BMI	26.6 ± 6	27.7 ± 6.3	0.72
Prealbumin (20–40 mg/dL)	8.6 ± 4.4	8.8 ± 4.3	0.41
Albumin (34–48 g/L)	29.7 ± 5.7	30 ± 5.9	0.89
Cholesterol (125–200 mg/dL)	114.3 ± 31.1	116.1 ± 55.2	0.78
Triglycerides (50–200 mg/dL)	80.8 ± 21.8	92.6 ± 56.1	0.9
Decompensation of cirrhosis			
Ascites	25 (78.1%)	72 (82.8%)	0.59
SBP	4 (12.5%)	7 (8%)	0.48
Encephalopathy	11 (34.4%)	17 (19.5%)	0.14
Portal hypertensive bleeding	4 (12.5%)	13 (14.9%)	0.49
Non-SBP infection	11 (34.4%)	29 (33.3%)	0.54
Alcoholic hepatitis	2 (6.3%)	15 (17.2%)	0.15
AKI	3 (9.4%)	11 (12.6%)	0.75
Vitamin A levels (0.3–1 mg/L)	0.11 ± 0.1	0.13 ± 0.12	0.63
Vitamin A deficiency (<0.3 mg/L)	30 (96.8%)	79 (91.9%)	0.67
Vitamin D levels (>30 ng/dL)	9.5 ± 5.5	8.9 ± 6.8	0.15
Vitamin D deficiency (<30 ng/dL)	26 (96.3%)	75 (98.7%)	0.44
Vitamin E levels (5–20 µg/dL)	8.9 ± 3.6	8.5 ± 3.2	0.5
Vitamin E deficiency (<5 µg/dL)	4 (12.9%)	13 (15.1%)	0.76
Vitamin K levels (0.13–1.50 µg/L)	0.64 ± 0.4	0.88 ± 0.8	0.52
Vitamin K deficiency (<0.13 µg/L)	0	3 (5.7%)	0.56
Vitamin B1 levels (2–7.2 µg/dL)	4.1 ± 1.3	3.9 ± 1.9	0.51
Vitamin B1 deficiency (<2 µg/dL)	0	3 (5.7%)	9.56
Vitamin B6 levels (23–173 nmol/L)	27.8 ± 21.5	25.6 ± 20.4	0.9
Vitamin B6 deficiency (<23 nmol/L)	17 (54.8%)	51 (61.4%)	0.53
Vitamin B9 levels (2–14.54 ng/mL)	7.1 ± 4.7	6.8 ± 4.2	0.88
Vitamin B9 deficiency (<2 ng/mL)	1 (4.3%)	4 (5.7%)	0.8
Vitamin B12 levels (150–695 pg/mL)	1076.6 ± 611.9	1058 ± 606.8	0.29
Vitamin B12 deficiency (<150 pg/mL)	0	0	1
Calcium (8.8–10.2 mg/dL)	9.3 ± 0.4	9.3 ± 0.5	0.57
Calcium deficiency (<8.8 mg/dL)	0	6 (7.5%)	0.13
Phosphorous (2.7–4.5 mg/dL)	3 ± 0.7	2.7 ± 0.6	0.58
Phosphorous deficiency (<2.7 mg/dL)	8 (28.6%)	33 (41.8%)	0.26
Magnesium (1.6–2.6 mg/dL)	1.9 ± 0.34	1.9 ± 0.41	0.89
Magnesium deficiency (<1.6 mg/dL)	3 (13%)	12 (16.9%)	0.66
Copper (70–140 µg/dL)	83.6 ± 31.2	98.4 ± 34.4	0.43
Copper deficiency (<70 µg/dL)	6 (20%)	12 (18.8%)	0.88
Iron (60–158 µg/dL)	87.4 ± 7.1	70.4 ± 53.6	0.84
Iron deficiency (<60 µg/dL)	13 (44.8%)	33 (39.3%)	0.6
Ferritin (30–400 ng/mL)	275.8 ± 323.6	418.7 ± 741.9	0.44
Ferritin deficiency (<30 ng/mL)	1 (3.7%)	4 (4.8%)	0.81
Zinc (68–120 µg/dL)	45.4 ± 15.9	44.8 ± 18.8	0.76
Zinc deficiency (<68 µg/dL)	27 (93.1%)	74 (89.2%)	0.54

Data are mean ± SD for quantitative variables and n (%) for qualitative variables. MELD: Model for end-stage liver disease; SBP: Spontaneous bacterial peritonitis; BMI: Body mass index; AKI: Acute kidney injury.

## Data Availability

The datasets generated and analyzed during the current study are not publicly available due to provisions of the written informed consent.
